# *Pllans−II*: Unveiling the Action Mechanism of a Promising Chemotherapeutic Agent Targeting Cervical Cancer Cell Adhesion and Survival Pathways

**DOI:** 10.3390/cells12232715

**Published:** 2023-11-27

**Authors:** Alejandro Montoya-Gómez, Fiorella Tonello, Barbara Spolaore, Maria Lina Massimino, Leonel Montealegre-Sánchez, Andrés Castillo, Nelson Rivera Franco, María José Sevilla-Sánchez, Luis Manuel Solano-Redondo, Mildrey Mosquera-Escudero, Eliécer Jiménez-Charris

**Affiliations:** 1 Grupo de Nutrición, Facultad de Salud, Universidad del Valle, Cali 760043, Colombia; leonel.montealegre@correounivalle.edu.co (L.M.-S.); maria.sevilla@correounivalle.edu.co (M.J.S.-S.); lmsolanor@gmail.com (L.M.S.-R.); mildrey.mosquera@correounivalle.edu.co (M.M.-E.); 2 Istituto di Neuroscienze, CNR, Via Ugo Bassi 58/B, 35131 Padova, Italy; fiorella.tonello@cnr.it (F.T.); marilina.massimino@gmail.com (M.L.M.); 3 Dipartimento di Scienze del Farmaco, Università di Padova, Via F. Marzolo 5, 35131 Padova, Italy; barbara.spolaore@unipd.it; 4 Grupo de Investigación en Ingeniería Biomédica-GBIO, Universidad Autónoma de Occidente, Cali 760030, Colombia; 5 TAO-Lab, Centre for Bioinformatics and Photonics-CIBioFi, Department of Biology, Universidad del Valle, Cali 760032, Colombia; andres.castillo.g@correounivalle.edu.co (A.C.); rivera.nelson@correounivalle.edu.co (N.R.F.)

**Keywords:** snake venom, phospholipase A_2_, bioprospecting, antitumor potential, transcriptomic analysis, membrane receptor target

## Abstract

Despite advances in chemotherapeutic drugs used against cervical cancer, available chemotherapy treatments adversely affect the patient’s quality of life. For this reason, new molecules from natural sources with antitumor potential and few side effects are required. In previous research, *Pllans−II*, a phospholipase A_2_ type-Asp49 from *Porthidium lansbergii lansbergii* snake venom, has shown selective attack against the HeLa and Ca Ski cervical cancer cell lines. This work suggests that the cytotoxic effect generated by *Pllans−II* on HeLa cells is triggered without affecting the integrity of the cytoplasmic membrane or depolarizing the mitochondrial membranes. The results allow us to establish that cell death in HeLa is related to the junction blockage between α_5_β_1_ integrins and fibronectin of the extracellular matrix. *Pllans−II* reduces the cells’ ability of adhesion and affects survival and proliferation pathways mediated by intracellular communication with the external environment. Our findings confirmed *Pllans−II* as a potential prototype for developing a selective chemotherapeutic drug against cervical cancer.

## 1. Introduction

In 2020, the International Agency for Research on Cancer (IARC) generated the latest estimate of incidence and mortality for the different types of cancer worldwide. In this study, cervical cancer (CC) was reported as the fourth most common cancer and the fourth-leading type of cancer in causing the most deaths in women of reproductive age around the world, with approximately 604,000 new cases and 342,000 deaths for that year [1,2]. The highest incidence and mortality rates for this type of cancer are observed in countries with the lowest human-development indices, despite global attempts to achieve early detection and effective vaccination against the human papillomavirus (HPV) [2,3,4]. In its early stages, the standard treatment for CC is still radical surgery. For patients with high-risk factors for progressing pathology, adjuvant radiotherapy should be administered with concurrent cisplatin-based chemotherapy [5,6]. However, it is widely recognized that these treatments substantially increase complications and side effects, such as lymphedema, sexual dysfunction, urinary frequency and bladder dysfunction, bowel dysfunction, and other symptoms derived from nephrotoxicity, ototoxicity, and neurotoxicity [4,7]. Therefore, finding more effective treatments for this pathology with better safety profiles and new modes of action is urgent.

Over the past three decades, research has shown that snake-venom proteins are promising candidates in drug designs for CC therapy [8,9,10,11,12]. Among these proteins, snake-venom phospholipases A_2_ (svPLA_2_), a group of enzymes that exert their catalytic action by lysing phospholipids, vesicles, or biological membranes [13], stand out for their marked cytotoxic effect against cancer cells, with little or no effect against non-tumorigenic cells [10,14,15,16,17]. However, the antitumor effect of svPLA_2_ seems to be independent of its catalytic activity. Instead, the possibility of a selective effect against cancer cells mediated through C-terminal region electrostatic interactions with cytoplasmic membrane components such as cell proliferation receptors, cell death receptors, or extracellular matrix (ECM) adhesion integrins has been postulated [8,10,14,18,19,20,21,22]. Another mechanism, demonstrated by Massimino et al., involves the interaction of a Lys49 svPLA_2_ from *Bothrops asper* with cell membrane nucleolin, which subsequently mediates the cellular enzymes’ internalization [23]. These events were related to the myotoxic effect induced by the svPLA_2_ and are probably related to the selectivity against tumor cells, since nucleolin overexpression and its increased localization at the cytoplasmic membrane level is a characteristic of several types of tumor cells [24]. However, most studies that associate svPLA_2_ with an antitumoral effect are based on findings with types of svPLA_2_ Lys-49, which, despite lacking catalytic activity, are recognized for high toxicity [25]. This characteristic could be counterproductive if the objective is to develop an innocuous drug against non-tumorigenic cells. Thus, our group has searched in recent years for bioactive molecules that selectively attack CC cells. Within the framework of this work, we have characterized the biological effect exerted by *Pllans−II*, a svPLA_2_ type Asp-49 from *Porthidium lansbergii lansbergii*, on CC tumor lines. These investigations have shown that *Pllans−II* generates a cytotoxic effect on cervical adenocarcinomaa cells (HeLa) and cervical squamous epithelial carcinoma cells (Ca Ski). Our experiments have also demonstrated that *Pllans−II* causes this effect on CC tumor lines without affecting either the viability of non-tumorigenic cell lines, such as MCF-10A (non-tumorigenic breast cells), HUVEC (human umbilical vein endothelial cells), and C2C12 (murine skeletal muscle C2C12 myotubes), or myotoxicity activity or Intracerebro-ventricular lethal activity on CD-1 mice [10,14,26]. However, although the effect induced by *Pllans−II* on both cell lines coincides with affecting adhesion, migratory potential, cell cycle arrest, and death by apoptosis, we only know the intracellular mechanism that affects *Pllans−II* against Ca Ski, which is related to the inability to resolve endoplasmic reticulum stress induced by sub-expression of genes such as PERK, ERO1, PDIs, HSP70, and CHOP [14]. Therefore, this study focused on determining the effects induced by *Pllans−II* in HeLa cells at the cytoplasmic membrane level and elucidating its anticancer mechanism.

## 2. Materials and Methods

### 2.1. Venom Collection and Pllans−II Purification

The snake venom from *Porthidium lansbergii lansbergii* was obtained from specimens collected in three municipalities of the Atlantic Department, Colombia: Juan de Acosta, Piojó, and Baranoa. The milking of the snake venom was performed by trained professionals using proper safety precautions. The ejected venom was collected in 1.5 mL vials, the snakes were released after venom collection, and all samples were pooled, centrifuged to remove debris, dried in a vacuum centrifuge, and stored at −20 °C until their use. Sample collection was allowed by Autoridad Nacional de Licencias Ambientales—ANLA, Resolution Nº 1070, 28 August 2015, and a contract for access to genetic resources and their derivative products, No. 307, 3 March 2021, was granted by the Ministerio de Ambiente of Colombia for the purposes of the present investigation.

Lyophilized venom pools were resuspended in H_2_O/0.1% trifluoroacetic acid (TFA) at 10 mg/mL, and *Pllans−II* protein was obtained using RP-HPLC in the same manner as described previously [10]. In brief, aliquots of venom (2 mg) were separated in a Zorbax SB-C_18_ column (250 × 4.6 mm, 5 μm particle diameter; Agilent) eluted at 1 mL/min with a gradient from H_2_O/0.1% TFA to acetonitrile/0.1% TFA. The eluent was monitored at 215 nm in an Agilent 1260 Infinity chromatograph using the EZ CHROM ELITE software, version 4.9.005.0 (Agilent Technologies, Inc. 2006, Santa Clara, CA, USA). The target svPLA_2_ fraction was collected manually, dried by vacuum centrifugation, and stored at −20 °C.

### 2.2. Cell Culture and IC_50_ Determination

The HeLa cell line was obtained from the American Type Culture Collection (ATCC CCL-2™; Manassas, VA, USA) and maintained at 37 °C in a humidified incubator containing a 5% CO_2_ atmosphere. EMEM media containing 10% FBS, 2 mM L-glutamine, 2 mM sodium pyruvate, 1 mM non-essential amino acids, 100 U/mL penicillin, and 100 mg/mL streptomycin were used to culture the cell line. When confluency was reached, the cells were divided for the experiments.

The IC_50_ determination on HeLa cells with *Pllans−II* after the purification process was obtained using the Dr fit software (V. 1.042), following the procedure described by di Veroli et al. [27].

### 2.3. Proliferation Assay

The HeLa cells proliferation treated with *Pllans−II* was evaluated by the MTT assay (Roche Diagnostics, Mannheim, Germany, catalog number 11465007001) as described previously [15,16]. Briefly, cells (1.5 × 10^4^ cells/well) were seeded in 96-well microplates and incubated for 24 h. After adhesion, cells were treated with *Pllans−II* (12.5, 25, 50, and 100 μg/mL) or EMEM medium supplemented with FBS as a control for 24, 48, and 72 h at 37 °C in 5% CO_2_. After treatment, the cells were incubated with MTT (3-(4,5-dimethylthiazol-2-yl)-2,5-diphenyl tetrazolium bromide) (5 mg/mL, 20 μL/well) for 3 h at 37 °C, and the formazan crystals were dissolved in 100 μL of 10% SDS and 0.01 M HCl, followed by incubation for 18 h at 37 °C and 5% CO_2_. The absorbance was read after 18 h on a multi-well scanning spectrophotometer at 570 nm in a microplate reader (iMark^®^ Microplate Absorbance Reader–BioRad, Hercules, CA, USA). The experiment was carried out in triplicate for each time-point and each concentration.

### 2.4. Visualization of HeLa Morphological Alterations Induced by Pllans−II

The morphological alterations in HeLa cells after exposure to *Pllans−II* were observed by scanning electron microscopy (SEM), as described by Möbius et al. [28], with slight modifications. Briefly, cells (7 × 10^5^/well) were seeded in circular glass plates, immersed in culture-plate wells, and kept at 37 °C with 5% CO_2_ for 24 h. After this, the cells were incubated for 24 h with the culture medium in the absence (control) or presence of *Pllans−II* inhibitory concentration 50 (IC_50_, 100 μg/mL). Then, the cells were fixed with glutaraldehyde, formaldehyde, and phosphate buffer and dehydrated with different ethanol concentrations (20, 40, 60, 80, 90, and 100%). Finally, the samples were dried at room temperature, bathed in a thin layer of plasma-induced gold, and analyzed in a NeoScope JCM-5000 (NIKON/JEOL, Tokyo 196-8558, Japan).

### 2.5. Mitochondrial Membrane Potential Analysis

The change in mitochondrial membrane potential (Δψm) in HeLa cells was determined using the Mitochondrial Membrane Potential Detection Kit (Agilent Technologies, La Jolla, CA, USA, catalog number 280002), following the manufacturer’s protocol. Briefly, cells (5 × 10^5^/well) were seeded in 12-well culture plates and kept at 37 °C with 5% CO_2_ for 24 h. After this, the cells were incubated in triplicate with medium, either in the absence (control) or in the presence of an IC_50_ of *Pllans−II*, for 24 h.

Later, the cells were resuspended in 1X buffer solution with the JC-1 probe and incubated at 37 °C with 5% CO_2_ for 15 min, to be later washed and centrifuged. Finally, the pellet was resuspended in a 1X assay buffer. The percentage of cells with the probe in aggregated/monomeric form was determined by flow cytometry (BD Accuri ™ C6–Biosciences, San José, CA 95131, USA).

For the visualization of the cells in a confocal microscope (Zeiss Axio Scope A1, LSM 700), the culture medium was removed after the treatment with the protein or control, and 500 µL of 1X buffer solution with the JC-1 probe was added for incubation at 37 °C with 5% CO_2_ for 15 min. The JC-1 reagent was removed, and the cells were washed once with 1X assay buffer. After removing the assay buffer, the cells were covered with a coverslip. Immediately, the cells were analyzed in a confocal microscope using a bandpass filter to detect JC-1 aggregates (excitation/emission = 590/610 nm) or JC-1 monomers (excitation/emission = 490/520 nm). JC-1 forms J-aggregates in healthy mitochondrial matrices, which can be visualized as red fluorescence. In depolarized mitochondria, JC-1 effluxes to the cytoplasm and generates a green fluorescence [29,30,31]. Confocal microscopy images were acquired and analyzed with the Leica LAS AS software, version 2.7.0.

### 2.6. Lactate Dehydrogenase (LDH) Assay

To detect whether the cell membrane was damaged by treatment with *Pllans−II*, LDH release into the culture medium was measured. Briefly, HeLa cells (1.5 × 10^4^/well) were seeded in 96-well plates and incubated with an IC_50_ of *Pllans−II*, culture medium (control) or Triton X-100 (positive control) at 37 °C with 5% CO_2_ for 24 h. After incubation, 50 μL of the medium was collected, and LDH activity was quantified using the LDH Cytotoxicity Assay Kit following the manufacturer’s protocol (Sigma-Aldrich, Catalog Number MAK066, Billerica, MA, USA). LDH activity was quantified by considering that one unit of LDH activity is defined as the amount of enzyme that catalyzes the conversion of lactate into pyruvate to generate 1.0 μmole of NADH per minute at 37 °C. Therefore, the formazan produced in this reaction was proportional to the quantity of LDH released into the culture medium resulting from cytotoxicity, and was measured on a microplate reader (iMark^®^ Microplate Absorbance Reader–BioRad, Hercules, CA, USA) at 450 nm.

### 2.7. Three-Dimensional Structure of Pllans−II and Molecular Docking with α_5_β_1_ Integrin

*Pllans−II* was alkylated, reduced, and digested with the Proteo Extract All-in-One Trypsin Digestion Kit (Merck Millipore, Billerica, MA, USA, catalog number 650212). In accord with the manufacturer’s instructions, to obtain the highest possible sequence coverage, the protein was additionally digested with chymotrypsin and Glu-C (both from Promega, Madison, WI, USA). The reverse-phase nano-HPLC was used to separate the desalted peptides (Dionex Ultimate 3000, Thermo Fisher Scientific, Bremen, Germany). The column (Acclaim PepMap RSLC C_18_, 75 μm × 15 cm, Dionex, Thermo Fisher Scientific, Bremen, Germany) was developed with an acetonitrile gradient (solvent A: 0.1% formic acid; solvent B: 0.1% formic acid/90% acetonitrile; 5–45% B in 120 min) at a flow rate of 300 nL/min at 55 °C. The samples were analyzed in a Q Exactive Orbitrap mass spectrometer (Thermo Fisher Scientific, Bremen, Germany). The capillary voltage of the nanospray was 2 kV. Lock mass calibration was used for the highest accuracy. Peptide fragmentation/identification was made with normalized fragmentation energy at 27%. Sequence assignment was performed with PEAKS Studio 8.5 (Bioinformatics Solutions, Waterloo, ON, Canada) using a combination of de novo sequencing and database search (UniProt, www.uniprot.org). Only de novo sequenced peptides with high average local confidence, (ALC) ≥ 80%, including FDR ≤ 1%, were accepted. High-quality de novo sequence tags were then used by PEAKS DB to search the entire *Swiss-Prot* database.

The 3D model of *Pllans−II* was generated by sequence homology utilizing SWISS-MODEL, accessible through the Expasy server (https://swissmodel.expasy.org, accessed on 12 February 2023), using the sequence template of an acidic phospholipase A_2_ from *Deinagkistrodon acutus* [32]. This model was evaluated with the QMEAN function of the SWISS-MODEL server [33]. Subsequently, we determined the interactions of *Pllans−II* and α_5_β_1_ integrin ectodomain, a transmembrane receptor that may be related to apoptosis, adhesion, and migratory capacity, as reported previously [10,14]. Briefly, the three-dimensional structure of the α_5_β_1_ integrin ectodomain was obtained in the protein-data-bank repository (PDB ID: 3VI3) and modified with the UCSF Chimera software (1.14, 2023), eliminating the elements that did not correspond to the ectodomain of the protein (FAB and glucopyranose). Molecular docking between *Pllans−II* and the integrin α_5_β_1_ was simulated using the ClusPro version 2.0 server (https://cluspro.org/, accessed on 3 March 2023) [34]. Additionally, the interaction between α_5_β_1_ integrin and fibronectin (PDB ID: 3M7P), a physiological integrin ligand reported in the literature [35], was modeled. The docking with the best “efficient global docking value” was selected for each pair of coupled proteins. The Pymol (version 2.0) software (https://pymol.org/2/, accessed on 25 March 2023) and UCSF Chimera (1.14, 2023) were used to visually represent and identify the interactions between the receptor amino acids and *Pllans−II* at a distance equal to or less than 3Å. Further, the PDBsum server tool (http://www.ebi.ac.uk/thornton-srv/databases/pdbsum/, accessed on 1 May 2023) was used to determine the residue-to-residue interactions between *Pllans−II*-α_5_β_1_ integrin and fibronectin-α_5_β_1_ integrin.

### 2.8. Pllans−II Derivatization and Visualization of Interactions with HeLa Cells

To determine the cellular compartments in which *Pllans−II* interacts with HeLa cells, *Pllans−II* was derivatized with FITC (Thermo Fisher Scientific, OR, USA, catalog number F1906) to put it in contact with HeLa cells, and the interactions were observed by confocal microscopy, according to the experimental design developed by Dutta et al. [36], with some modifications. For the derivatization process, *Pllans−II* (2 mg/mL) was dissolved in 0.1 M sodium bicarbonate buffer at pH 9.0 and incubated with FITC for 1 h at room temperature with constant agitation (Protein:FITC ratio of 1:2 molar). Then, hydroxylamine (1:10 *v*/*v* ratio) was added to the reaction mixture, with an incubation time of 1 h at room temperature, to stop the reaction. Next, the derivatized product (*Pllans−II*-FITC) was dialyzed using cellulose membrane (Serva Membra-cell dialysis tubing, diameter 16 mm, catalog number 44310.02) and acidified with 5% trifluoroacetic acid for subsequent purification by RP-HPLC on an Avantor-C_18_ column (150 × 4.6 mm, 5 μm particle diameter). Elution was performed at 1 mL/min by applying a gradient from solution A (H_2_O/0.1% TFA) to solution B (acetonitrile/0.1% TFA) as follows: 5% B for 3 min, 5–30% B over 3 min, 30–44% B over 14 min, 44–95% B over 1 min, 95% B for 3 min, 95–5% B over 1 min and 5% B over 7 min, reading the absorbance at 226 nm. The collected fractions were lyophilized and stored at −20 °C until use. Mass spectrometry analyses of derivatized products were performed using a Micromass Q-Tof micro (Waters, Milford, MA, USA). Instrument control and data acquisition and processing were performed with Masslynx software, version 4.2 (Micromass, Wilmslow, UK).

To verify the conservation of the enzymatic activity of *Pllans−II*-FITC, an in vitro assay was developed on the synthetic monodisperse substrate NOBA according to the Holzer and Mackessy method [37]. Briefly, 25 μL of various amounts of *Pllans−II*-FITC were mixed in 96-well plates, together with 200 μL of 10 mM Tris; 10 mM CaCl_2_; 0.1 M NaCl, pH 8.0; and 25 μL NOBA (Thermo Fisher Scientific, MA, USA, catalog number J67360) to achieve a NOBA concentration of 0.32 mM. The plates were incubated at 37 °C for 60 min, and absorbances were recorded at 405 nm on a Microplate Spectrophotometer plate reader (IMARK, Bio-Rad). Phospholipase activity was expressed as the change in absorbance × 1000.

*Pllans−II*-FITC (80 μg/mL) was added to the growth medium of HeLa cells, which were previously seeded (3 × 10^4^ cells/well) and cultured in circular cover glasses inside 24-well plates. The HeLa cells were incubated with FITC alone and saline solution as control treatments. After incubation, the culture medium was removed, and the cells were gently washed with saline solution and fixed by adding 2% paraformaldehyde (PFA) and incubating at 4 °C for 20 min. Then, the PFA was removed, and the cells were washed with PBS and incubated for 15 min at room temperature with 0.1% Triton-X100 for cell permeabilization. Once the cells were permeabilized, the Triton was removed, and the cells were washed with PBS and incubated with HOECHST 33342 (5 µg/mL) (Sigma-Aldrich, Irvine, UK, catalog number H6024) at *v*/*v* ratio 1:1000 for 15 min at room temperature in the dark for nuclear staining. Finally, the glass slides were mounted in 8% Mowiol 40–88 (Sigma-Aldrich, Saint Louis, MO, USA, catalog number 324,590) in glycerol and PBS (1:3 = *v*/*v*) and visualized under a confocal microscope system (Leica TCS-SP5). Confocal microscopy images were acquired and analyzed with the Leica LAS AS software, version 2.7.0. Confocal reported images are a single plane of a complete z-stack of the observed field, composed of 10–15 sections taken with a step-size of 0.15–0.20 µm, pinhole [m] 67.9 µm, pinhole [airy] 1.00.

### 2.9. Adhesion Inhibition Evaluation

According to the Varol protocol [38], 50 µL/well of human fibronectin (10 µg/mL in PBS) (Gibco™, Carlsbad, ON, Canada, catalog number 33016015) or ECM gel from Engelbreth–Holm–Swarm murine sarcoma (Sigma-Aldrich, Saint Louis, MO, USA, catalog number E6909) (1 mg/mL in PBS) was incubated for 12 h at 4 °C in 96-well plates. After incubation, the remaining liquid was removed, and each well was washed three times with 100 µL of 1X PBS and allowed to dry for 1 h at room temperature. During plate drying, HeLa cells (2 × 10^4^ cells/100 µL) were pre-incubated in triplicate with the IC_50_ of *Pllans−II* or with culture medium (control) for 1 h at 37 °C in a humidified environment with 5% CO_2_. After the pre-incubation, the cells were seeded in the wells treated with fibronectin or Matrigel and left to incubate for 3 h at 37 °C in a humidified environment with 5% CO_2_. Non-adhered cells were removed with three washes of 100 µL of 1X PBS. Next, 100 µL of culture medium and 20 µL of MTT (5 mg/mL) were added to each well. After incubating for 3 h at 37 °C, 100 µL of 1X PBS with 10% SDS and 0.01M HCl was added to each well and incubated for 18 h at 37 °C in 5% CO_2_. Finally, absorbances at 570 nm were read on a Microplate Spectrophotometer plate reader (IMARK, Bio-Rad). The inhibition of cell adhesion was expressed as the average of the absorbance values obtained in the treatments in triplicate in contrast to the absorbance of the control group.

Additionally, by adding *Pllans−II*, it was determined whether the HeLa cells that had adhered to wells pre-treated with fibronectin or Matrigel interrupted interactions between cells and ECM proteins. The same pre-treatment was performed on the 96-well plates with fibronectin or Matrigel previously described, and HeLa cells were seeded (2 × 10^4^ cells/100 µL) in triplicate. The cells were incubated for 3 h at 37 °C in a humidified environment with 5% CO_2_. After incubation, the culture medium was removed, and 100 µL of medium with the IC_50_ of *Pllans−II* was added to each well to be incubated again for 3 h at 37 °C in a humidified environment with 5% CO_2_. After incubation, the same steps described above were followed to assess the viability of the adhered cells.

### 2.10. Transcriptomic Analysis

The HeLa cells (7 × 10^5^/well) were seeded in 12-well plates and kept at 37 °C with 5% CO_2_ for 24 h. After this, the cells were incubated for 24 h with the medium in the absence (control) or presence of *Pllans−II* (100 μg/mL). Total RNA was extracted using the RNeasy Micro kit (Cat. No. 74004). The extracted RNA’s purity, integrity, and potential contamination were verified in a Nanodrop 2000 (Thermo Scientific, Waltham, MA, USA) (OD 260/280), using bioanalyzer Agilent 2100 and agarose gel. The sample selection, cDNA synthesis, double-stranded cDNA purification, hybridization, PCR amplification, purification of PCR library products, sequencing, mapping of readings, and quantification of expressions were performed according to the method described by Montoya-Gómez et al., 2022 [14].

For the DEG pathway and functional enrichment analysis, the R cluster Profiler package [39] was used to identify the biological implications of DEG with the *Gene Ontology* database (GO, http://geneontology.org, accessed on 25 November 2022), within the category of biological processes (BP). In addition, a pathway analysis was performed with the *Kyoto Encyclopedia of Genes and Genomes* (KEGG, https://www.genome.jp/kegg/, accessed on 25 November 2022) database to identify biologically relevant pathways associated with DEG. Fisher’s exact test was used to select significant pathways based on adjusted *p* values, and those with a value < 0.05 were considered significant. From the 30 most significant enriched terms, an enrichment map was obtained with the enrich plot package of R, in which the sets of DEG shared between each pair of terms are related by lines, facilitating the identification of functional modules.

### 2.11. Statistical Analysis

All experiments were performed in triplicate. The results were expressed as mean ± SD. Student’s *t*-test or one-way ANOVA determined the difference significance between the means of treatment and control. In contrast, the comparison of two or more variables was assessed by the two-way ANOVA, followed by Bonferroni post-test, using the software GraphPad Prism version 5 (GraphPad Software, Inc., San Diego, CA, USA), where *p* < 0.05 was considered significant.

## 3. Results

### 3.1. Pllans−II Affects HeLa Cell Proliferation and Triggers Apoptosis

**Figure 1:**
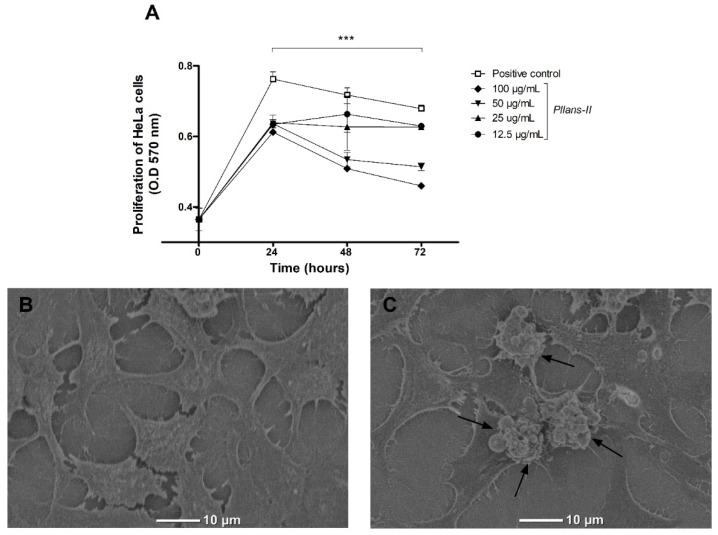
*Pllans−II* induces anti-proliferative effect and apoptotic death in HeLa cells. (**A**) Anti-proliferative effect of *Pllans−II* on Hela cell at 24, 48, and 72 h post-treatment. HeLa cells were treated with *Pllans−II* (12.5, 25, 50, and 100 μg/mL) vs. positive control (culture medium supplemented with SFB). The cell’s viability was evaluated by MTT. Data are expressed as mean ± SD, and procedures were carried out in triplicate. Statistically significant differences are observed with *** *p* < 0.001. (**B**) Analysis by scanning electron microscopy (SEM) of HeLa cells incubated in culture medium, in contrast to (**C**) HeLa cells treated with 100 μg/mL of *Pllans−II*. The apoptotic bodies of HeLa cells treated with *Pllans−II* are marked with black arrows.

The inhibition proliferation effect of *Pllans−II* on HeLa cells was evaluated at different concentrations by the MTT assay (Figure 1A). *Pllans−II* displayed a proliferative decrease on tumor cells for all concentrations evaluated, being more significative at 50 and 100 µg/mL. The highest concentration displayed a proliferation decrease of approximately 18%, 27%, and 32% at 24, 48, and 72 h, respectively.

Through SEM analysis, it was possible to visualize the formation of apoptotic bodies in the cytoplasmic membranes of HeLa cells treated with *Pllans−II* (Figure 1C), which were not observable in cells treated with culture medium (Figure 1B). Additionally, a change in cell morphology was evident, since many of the treated cells presented variations in shape and size, going from being polygonal and large to presenting a reduced size and a rounded shape.

### 3.2. Pllans−II Does Not Affect the Cytoplasmic Membrane or Mitochondrial Integrity

**Figure 2:**
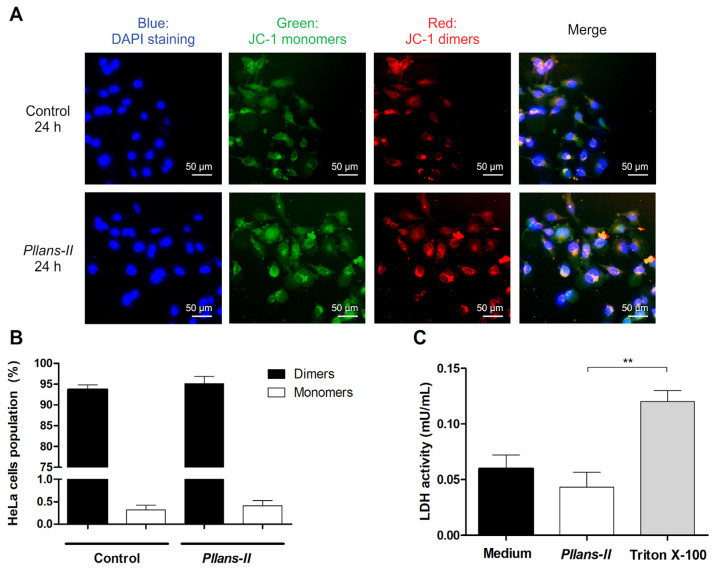
The effect induced by *Pllans−II* on HeLa cells is not related to alteration of the mitochondrial membrane integrity or damage to the cytoplasmic membrane. The effect of *Pllans−II* on the mitochondrial membrane potential of HeLa cells was evaluated by staining with a JC-1 probe after incubating the cells with 100 µg/mL of *Pllans−II* vs. EMEM medium as negative control. The cells were analyzed by (**A**) fluorescence microscopy and (**B**) flow cytometry. (**A**) The cells of the control group are shown in the upper four squares, and the lower four squares display the cells treated with 100 µg/mL of *Pllans−II*. The four squares display the cells treated with 100 µg/mL of *Pllans−II*. The blue color indicates the staining of cell nuclei with DAPI dye, the green color corresponds to the probe in its monomeric/disaggregated form, and the red/orange color corresponds to the probe in its dimeric form. (**B**) The percentages of HeLa cells with JC-1 probe in monomeric and dimeric form, respectively, for each treatment are shown. No statistically significant differences were observed between the treatments. (**C**) Effect of *Pllans−II* (100 μg/mL) on lactate dehydrogenase (LDH) levels in HeLa cell medium after 24 h of treatment, in contrast to negative and positive controls. Data are expressed as mean ± SD, and procedures were carried out in triplicate. Statistically significant differences are observed with ** *p* < 0.01.

The Δψm in HeLa cells treated with *Pllans−II* was evaluated by confocal microscopy (Figure 2A) and flow cytometry (Figure 2B) using the JC-1 probe. The analysis by both techniques revealed no differences between treatments for the JC-1 probe in its monomeric form and its dimeric form inside the cells evaluated. In cells treated with the svPLA_2_ and controls, the probe was found mainly in its dimeric form, indicating that the mitochondrial integrity was unaffected by the *Pllans−II* treatment. On the other hand, the *Pllans−II* affectation analysis of the integrity of the cytoplasmic membrane revealed that the treatment with the protein did not increase the release of the LDH enzyme (Figure 2C), which indicates that *Pllans−II* does not permeabilize the membrane.

### 3.3. Pllans−II Interacts with HeLa Cells at the Cytoplasmic Membrane Level, Disrupting Adhesion Interactions with ECM Proteins

**Figure 3:**
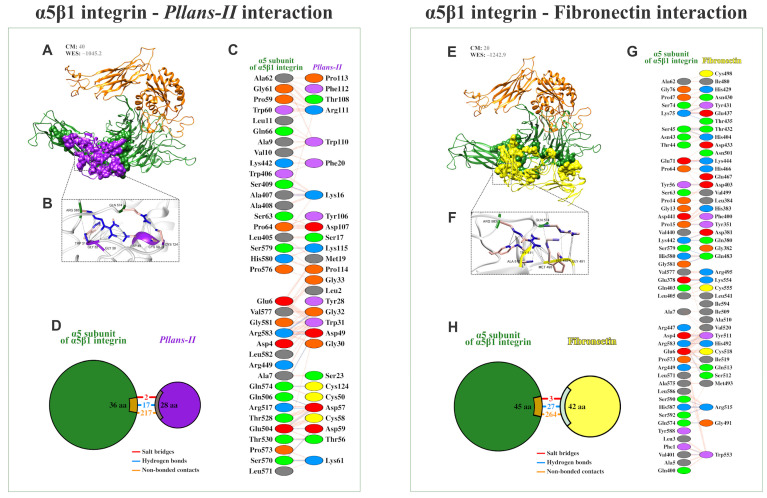
*Pllans−II* shows a high potential for interaction with the integrin α_5_β_1_ and in competing with fibronectin for the binding site. (**A**) Ribbon diagram of the most favorable docking models predicted by the ClusPro 2.0 server for determining the interaction of the α_5_β_1_ integrin (α chain in green, β chain in orange) with *Pllans−II* (purple). At the top are shown the cluster members (CM), the close neighborhoods that contribute most significantly to the favorability of the interaction between receptor and ligand, and the weighted energy score (WES) of the interaction. (**B**) Amplification of some close interactions between α_5_β_1_ integrin and *Pllans−II* amino acids residues. (**C**) Detail of interacting residues of α_5_β_1_ integrin and *Pllans−II*. The number of lines indicates the potential number of bonds. For non-bonded contacts, the width of the striped line indicates the number of potential contacts. (**D**) The pie chart shows the key interactions of residues between α_5_β_1_ integrin and *Pllans−II*. The key interactions are color-coded as follows: salt bridge (red), hydrogen bonds (blue), and non-bonded contacts (orange). (**E**) Ribbon diagram of the most favorable docking models predicted for the interaction of the α_5_β_1_ integrin (α chain in green, β chain in orange) with fibronectin (yellow). (**F**) Amplification of some close interactions between α_5_β_1_ integrin and *Pllans−II* amino acids residues. (**G**) Detail of interacting residues of α_5_β_1_ integrin and fibronectin. (**H**) The pie chart shows the key interaction of residues between α_5_β_1_ integrin and fibronectin.

The tryptic digestion and mass spectrometry analysis of *Pllans−II* allowed us to obtain the protein’s complete sequence of amino acids. The protein’s three-dimensional structure was modeled with this data, and the *Pllans−II* interaction with the α_5_β_1_ integrin (a transmembrane protein) was evaluated in silico. The analysis showed that *Pllans−II* has a high energetic favorability to interact with α_5_β_1_ (Figure 3A, WES: −1045.2). Although this value of energetic favorability was not higher than that of the α_5_β_1_ interaction with its physiological ligand—fibronectin (Figure 3E, WES: −1242.9), the number of clusters was higher for the *Pllans−II*—integrin compared to the integrin—fibronectin interaction (CM: 40 vs. 20). Interestingly, both models showed that several α_5_β_1_ amino acids, with which both *Pllans−II* and fibronectin amino acids had close interactions, were the same (Figure 3C,G). The PDBsum server predicted 236 interactions between 28 amino acids of *Pllans−II* and 36 amino acids α_5_β_1_ integrin (Figure 3C,D), and 294 interactions between 42 amino acids of fibronectin and 45 amino acids of α5β1 integrin (Figure 3G,H). Of the two hundred thirty-six interactions between *Pllans−II* and α_5_β_1_ integrin, two corresponded to salt bridges, seventeen to hydrogen bonds, and two hundred seventeen to non-bonded contacts (Figure 3D). On the other hand, of the two hundred ninety-four interactions between fibronectin and α_5_β_1_ integrin, three corresponded to salt bridges, twenty-seven to hydrogen bonds, and two hundred sixty-four to non-bonded contacts (Figure 3H). There were amino acids from the integrin that coincided in presenting three or more interactions with amino acids from both ligands (Figure S1).

The *Pllans−II* derivatization with FITC was successful, as demonstrated by the reaction mixture’s RP-HPLC chromatographic profile and the products’ mass spectrometry analysis (Figure S2). The theoretical average mass obtained from the sequence was 13,952.8 Da. The mass spectrum of intact *Pllans−II* indicates the presence of three isoforms with slight mass differences (13,954.2 ± 0.16 Da; 13,896.1 ± 0.05 Da; 13,796.14 ± 0.04 Da) which, after conjugation with FITC, each showed an increase in mass of approximately 389 Da. This mass increase corresponds to the derivatization with one FITC molecule, indicating the mono-derivatization of each *Pllans−II* isoform. Additionally, the FITC-derivatized protein retained enzymatic activity without presenting statistically significant differences compared to the native protein (Figure S3). It should be noted that the enzymatic site-specific conjugation with microbial transglutaminase was also attempted. Still, it did not produce any derivatization in the case of *Pllans−II*, probably due to the absence of reactive lysine or glutamine residues [40].

**Figure 4:**
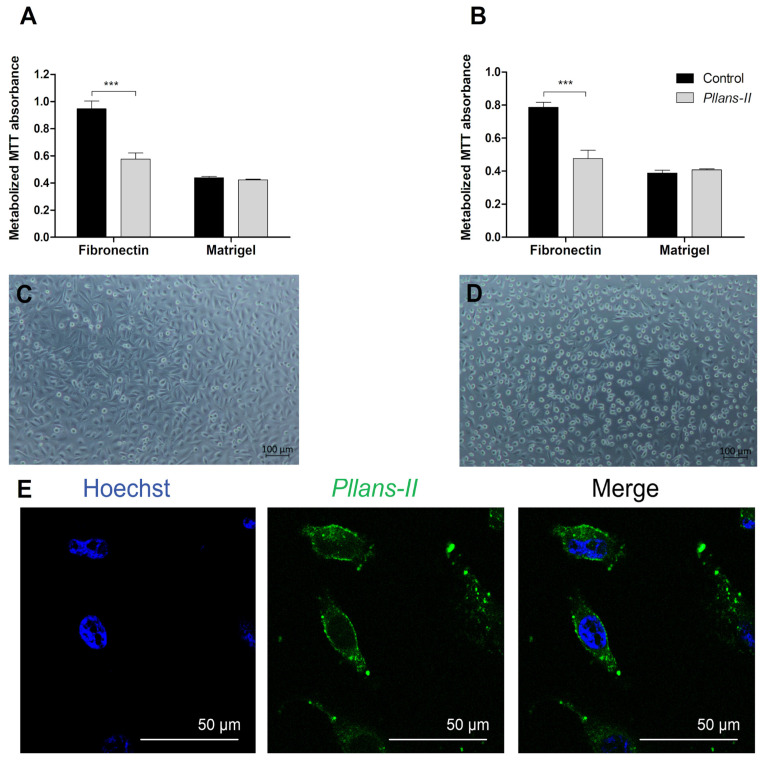
*Pllans−II* interacts with HeLa cells’ cytoplasmic membranes and affects their adhesion to fibronectin. (**A**) Evaluation of adhesion activity to HeLa cells pre-incubated for one hour with *Pllans−II* (100 µg/mL) or in EMEM culture medium (control). The cells were seeded in wells previously coated with components of the extracellular matrix: fibronectin or Matrigel. (**B**) Evaluation of adhesion inhibition on HeLa cells treated with *Pllans−II* (100 µg/mL) and control after being grown in wells previously coated with fibronectin or Matrigel. In (**A**,**B**), data are expressed as mean ± SD, and procedures were carried out in triplicate. Statistically significant differences are observed with *** *p* < 0.001. (**C**) HeLa cells were pre-incubated in culture medium and seeded in wells previously coated with fibronectin. (**D**) HeLa cells were pre-incubated with *Pllans−II* (100 µg/mL) for 1 h and seeded in a well previously covered with fibronectin. (**E**) Interaction of *Pllans−II*-FITC (Green, 80 μg/mL) with cytoplasmic membranes of HeLa cells exposed for 1 h, fixed, permeabilized with 0.5% Triton in PBS, and visualized by confocal microscopy. Nuclei were stained with Hoechst 33,342.

To validate the results obtained in the in silico and in vitro interaction analyses, the adhesion inhibition to substrates enriched either with fibronectin (Figure 4A) or with other ECM proteins (Figure 4B) was evaluated in HeLa cells treated with *Pllans−II*. In addition, adhesion inhibition was evaluated for both HeLa cells pre-treated with *Pllans−II* and those treated with *Pllans−II* after the adhesion process. The analyses revealed no statistically significant differences between cells treated with *Pllans−II* and the control group for both adhesion to Matrigel evaluations. However, for both evaluations, it was shown that *Pllans−II* significantly inhibits fibronectin adhesion, which could also be visualized under an inverted microscope, where it is evident that the cells of the control group do not present numerous detachments from the substrate (Figure 4C), contrary to findings with the *Pllans−II* treated cells (Figure 4D). Additionally, it was possible to show that the cellular compartment with which most interactions occur between HeLa cells and *Pllans−II*-FITC corresponds to the cytoplasmic membrane (Figure 4E).

### 3.4. Pllans−II Interaction with HeLa Cells Affects the Expression of Genes Mainly Related to Adhesion to the ECM and Proliferative Processes

**Figure 5:**
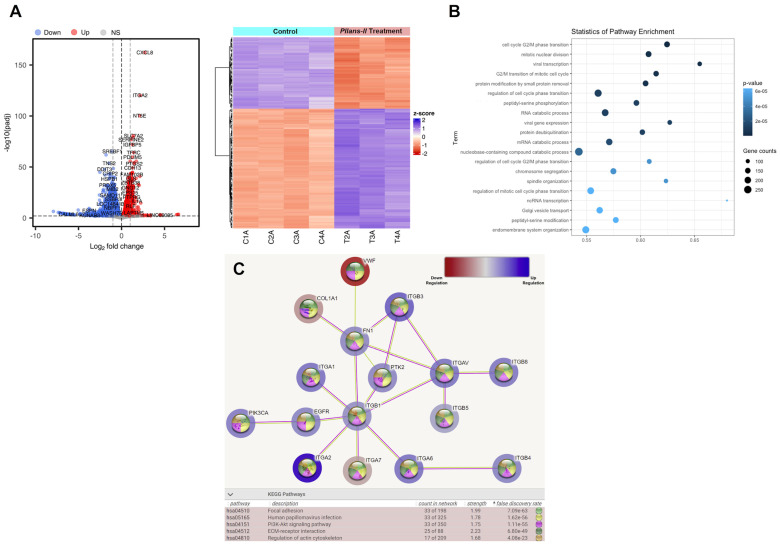
*Pllans−II* affects the expression of genes of proteins involved in the cell cycle, proliferation, and adhesion to ECM. (**A**) Analysis of differentially expressed genes (DEG) between HeLa cells treated with *Pllans–II*, and control: The red dots in the volcano plot represent genes with significantly higher expression (up–regulated) in the treatment. The blue dots represent down–regulated genes in the treatment group. The gray dots indicate genes not differentially expressed (NS: Non-significant). The heat map shows gene expression changes for each treatment and control sample. The red and blue colors represent the increase and decrease in expression, respectively, while the white color indicates no change. (**B**) Pathway enrichment analysis: The term (*Y*–axis) represents the pathway for the GO database. The enrichment factor (*X*–axis) represents the relationship between the differentially expressed genes and the total number of genes in each pathway. The area of each colored circle is proportional to the number of genes involved in each pathway; the color indicates the *p*–adjusted value. (**C**) The most affected functional module of enriched terms from HeLa cells treated with *Pllans−II*: The blue color indicates over-expression and the red under-expression. The spheres in each gene’s center indicate the biological pathways related to the affected gene. The color of the lines connecting the genes indicates the biological pathway associated with the affected genes. At the bottom, from left to right, are the KEGG codes for the altered biological pathways, the name of the pathway, the count of affected genes that are related to the biological pathway, the strength of the alteration to the pathway, and the color by which it is represented in the graph.

The difference in the gene expressions evaluated between the control and treatment groups is reported using a hierarchical grouping (heatmap) analysis and a volcano diagram (Figure 5A). *Pllans−II*-treated cells’ gene expressions were easily distinguished from those of control cells, suggesting a significant difference in gene expression between the two groups (Figure S4). A total of 6725 DEGs were identified. These included 3285 and 3440 downregulated and upregulated genes, respectively (Table S1). *Gene Ontology* (GO) analysis in the biological processes category (BP) showed 361 significantly enriched terms. Figure 5B shows the 20 most significantly enriched terms, with enrichment factor values between 0.53 and 0.73. The enriched biological processes include cell cycle regulation, chromosome segregation, mitotic nuclear division, protein removal pathways, mitotic spindle organization, and viral transcription. The analysis of the most affected biological pathways obtained from the KEGG database identifies alteration of adhesion pathways to the ECM, cytoskeleton regulation, the PI3K/AKT pathway of cell proliferation, and HPV infection. The overexpression of genes that code for integrins (ITGB3, ITGAV, ITGB8, ITGB5, ITGB1, ITGA1, ITGA2, ITGA6, ITGB4) is the factor that mainly explains the alteration of these pathways. However, there is also evidence of alteration in other essential genes in adhesion to the ECM and cancer cell proliferation, such as FN1, COL1A1, PIK3CA, PTK2, and EGFR (Figure 5C).

## 4. Discussion

Previous works carried out by our group have demonstrated the anticancer potential of *Pllans−II* on HeLa and Ca Ski cell lines, where evidence has suggested that the triggered cytotoxic effect is related to the induction of cell death by extrinsic pathways associated with transmembrane receptors rather than by intrinsic pathway apoptosis [10,14]. In this work, we add more evidence on the induction of apoptosis by *Pllans−II* on HeLa cells, observing the apoptotic bodies on the surfaces of the treated cells as visual markers of the cell death process. We also corroborate that the effect triggered in HeLa cells by *Pllans−II* is unrelated to mitochondrial membrane permeabilization, strengthening the hypothesis of an extrinsically mediated apoptotic effect reported previously by Jiménez-Charris et al. (2019), and, similar to what was observed in that study, down-regulation of genes involved in mitochondrial permeabilization, like bak and bax, was evidenced [10]. Additionally, we showed that *Pllans−II* generated a decrease in the proliferative capacity of HeLa cells, which is consistent with the affecting of the cell cycle, inhibition of adhesion and migration, and induction of apoptosis evidenced in previous studies on Ca ski and HeLa cells [10,14]. The effect on the cell cycle due to the action of *Pllans−II*, at least in HeLa cells, could be related to the alteration in the expression of kinase-dependent cyclins evidenced in our transcriptomic analysis, specifically the cyclins CDK17, CDK14, CDK6, CDK1, CDK8, CDK2AP1, CDK12, CDKL5, CDKN2AIP, CDKAL1, CDK13, and CDKN2B related to the G0/G1 and G2/M phases of the cell cycle. Additionally, we found that *Pllans−II* does not generate cytoplasmatic membrane damage in HeLa cells. Therefore, the initiation of the cell death signaling cascade could be caused by the activation of a transmembrane receptor. Evidence suggests the interactions of the C-terminal region of svPLA_2_s with these receptor types (i.e., integrins and growth-factor receptors), and these interactions may be related to the triggering of death in tumor cells [8,18,19,20,21,22].

For example, α_5_β_1_ integrin, the major ECM fibronectin receptor, is a protein shown to be overexpressed in CC cells and is associated with a poor prognosis [41,42]. The in silico modeling results demonstrated that the interaction between *Pllans−II* and the α_5_ subunit of the α_5_β_1_ integrin has a thermodynamic favorability close to that of the interaction between the integrin and its physiological ligand. Interestingly, the number of close interactions is similar for both models, and the interaction area with integrin is similar for both ligands, with the models even showing that several amino acids residues of the integrin α_5_ subunit are involved in the interaction with fibronectin and establish multiple interactions with *Pllans−II* residues as well. The *Pllans−II*—integrin interaction could affect the negatively charged Glu81, Glu124, and Asp154 surface amino acids residues of the α_5_ subunit. Despite these residues not directly interacting with fibronectin amino acids residues, they participate in creating the pocket for binding to RGD motifs [43]. This suggests a displacement for the physiological ligand interaction in the presence of *Pllans−II*, even when they have already adhered to fibronectin in in vitro tests. As support to the results obtained in in silico modeling, we found by fluorescence labeling that *Pllans−II* interacted, specifically, at the cytoplasmic membrane level, strengthening the interaction hypothesis with α_5_β_1_ integrins overexpressed in cytoplasmic membranes of CC cells.

Some studies on cancer-cell lines have shown that blocking the α_5_ subunit generates a cytotoxic effect [44]. For example, a study on the B16F10 melanoma cell line showed that the α_5_ subunit neutralization decreased cell adhesion, migration, and survival, inducing apoptosis and even reduced metastatic potential when evaluated on murine models [45]. In another study, the use of an anti-α_5_ antibody on three colon-cancer-cell lines (KM20, KM12C, and KML4A) reduced their adhesion capacity and induced apoptosis related to a decrease in the expression of phosphatidylinositol-3-kinase (PI3K) activity, the latter of which participates in cell proliferation and survival pathways [46]. The PI3K/Akt pathway disruption leaves cells susceptible to anoikis without survival signals [47,48,49,50].

We suggest that *Pllans−II* induces anoikis in Hela cells, since the affected gene expression is related to focal adhesion, PI3K/Akt signaling pathway, ECM-receptor interactions, regulation of actin cytoskeleton, cell division, and proliferation processes. Interestingly, many genes that code for integrins, protein tyrosine kinase 2 (PTK2), and fibronectin (FN1) were overexpressed. In the *Pllans−II* treatment context, this gene overexpression may be related to a compensatory activation mechanism of HeLa cells aiming to induce stress resistance, since, when a particular gene’s function in a network is disturbed, the expression of other genes within the same network may be altered as an attempt to maintain cellular wellness [51,52]. This strategy can work for the cell’s survival, given that most integrins play a redundant role in adhesion and signaling transduction. Notably, fibronectin overexpression plays a pivotal role in cellular adhesion and migration processes [53,54,55]. Additionally, the PTK2 gene overexpression, of a gene which codes for the focal adhesion kinase FAK1, may also correspond to a compensatory mechanism of this protein, normally overexpressed in CC cells. This can be a significant mediator of integrin-stimulated signal transduction pathways which would be altered with adhesion inhibition. Previous studies have revealed that the activation of PTK2 protects tumor cells via sustaining survival signaling [56]. The same compensatory mechanism could occur with the overexpression evidenced in the analysis for PIK3CA. This gene codes for the PI3K protein, an essential protein in the PI3K/AKT/mTOR signaling cascade that influences cell survival, growth, proliferation, and migration [57]. This pathway, like the HPV infection pathway, appeared enriched in the network of interactions obtained in the KEGG pathway. Both pathways are related to maintaining the immortal phenotype of CC cells, since the effective inhibition of senescence by the action of the E6/E7 oncoproteins requires mTORC1 activity [58].

Finally, in our study, an alteration in the expression of the EGFR gene was evidenced. This gene codes for the epidermal growth factor receptor protein, a tyrosine kinase family member related to the transformation of extracellular signals into critical responses for both epithelial cells’ normal development and the cancer cells’ progression to metastasis [59]. Interestingly, treatment with svPLA_2_s in cancer cells with a high EGFR density has affected their viability [8]. On the other hand, it has been shown that EGFR presents a cross-talk with ECM proteins, among them the α_5_β_1_ integrin. This interaction could modulate the EGFR signaling transmission [59,60]. For this reason, the *Pllans−II*—α_5_β_1_ integrin interaction could also affect EGFR signaling pathways related to cell viability and proliferation, and it is likely that the overexpression of the EGFR gene after treatment with *Pllans−II* also corresponds to a compensatory mechanism. However, according to the evidence of impaired viability and proliferation for CC cells treated with *Pllans−II*, they fail to survive treatment-induced stress, despite the compensatory upregulation mechanisms.

**Figure 6:**
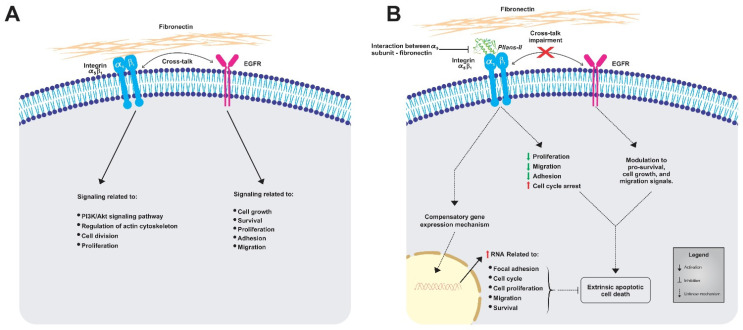
Representative model of the impacts generated by the alteration to interaction between α_5_β_1_ integrins from HeLa cells and fibronectin from ECM. (**A**) Representation of normal processes in untreated HeLa cells, where the α_5_β_1_ integrin-fibronectin and EGFR maintain signaling cascades for viability and metastatic potential. (**B**) Effects of *Pllans–II* on HeLa cells. The interference with α_5_β_1_ integrin-fibronectin interaction alters the intracellular signaling that maintains the immortal phenotype and the crosstalk between the integrin and the EGFR, triggering death by apoptosis via the extrinsic pathway.

Figure 6 summarizes the possible events triggered by *Pllans−II* in HeLa cells. We propose that when *Pllans−II* blocks the interaction between α_5_β_1_ integrin and ECM fibronectin, it generates alterations in the intracellular signaling that maintains the immortal phenotype of these cancer cells. *Pllans−II* could alter the crosstalk between the integrin and the EGFR, a function which also contributes to the modulation of cell survival, proliferation, and migration signals, triggering death by apoptosis via the extrinsic pathway. Additionally, as a consequence of the stress event, a compensatory mechanism is generated in cells that leads to increased expression of genes related to focal adhesion, cell cycle progression, proliferation, and migration, as an attempt to survive the events that lead cells to apoptosis.

## 5. Conclusions

This research showed that *Pllans−II* affects the viability and proliferative capacity of HeLa cells through a mechanism that does not compromise the integrity of the cytoplasmic membrane or affect the permeability of mitochondrial membranes. These CC cell alterations seem to be related to the junction blockage between α_5_β_1_ integrins and fibronectin of ECM. This reduces the cells’ ability of adhesion and affects survival and proliferation pathways mediated by intracellular communication with the external environment. The transcriptomic analysis suggests that due to *Pllans−II* treatment, HeLa cells develop compensatory gene upregulation mechanisms to survive the detachment which are unsuccessful, leading cells to a possible anoikis death process. Further studies with a proteomic approach and protein expression are required to understand the principal death intracellular pathways triggered by *Pllans−II* in CC cells.

## Data Availability

The data presented in this study are available in the article and the supporting information.

## Supplementary Materials

The following supporting information can be downloaded at: https://www.mdpi.com/article/10.3390/cells12232715/s1. Figure S1: α_5_β_1_ integrin amino acids residues with potential for multiple interactions with *Pllans−II* and fibronectin amino acids; Figure S2: RP-HPLC and mass spectrometry analyses of *Pllans−II* conjugation with FITC; Figure S3: FITC-derivatized *Pllans−II* retains its enzymatic activity; Figure S4: Quality control for transcriptomic analysis. Table S1: Raw data of differentially expressed genes after treatment of Hela cells with *Pllans−II*.
