# Supplementary material for: Pllans−II: Unveiling the Action Mechanism of a Promising Chemotherapeutic Agent Targeting Cervical Cancer Cell Adhesion and Survival Pathways
Source: Cells. 2023 Nov 27;12(23):2715. doi: 10.3390/cells12232715 (PMC10705806; doi:10.3390/cells12232715)
Supplement: Supplementary file 1 [file cells-12-02715-s001.zip › cells-2630276-supplementary figures.pdf]

Supplementary Materials

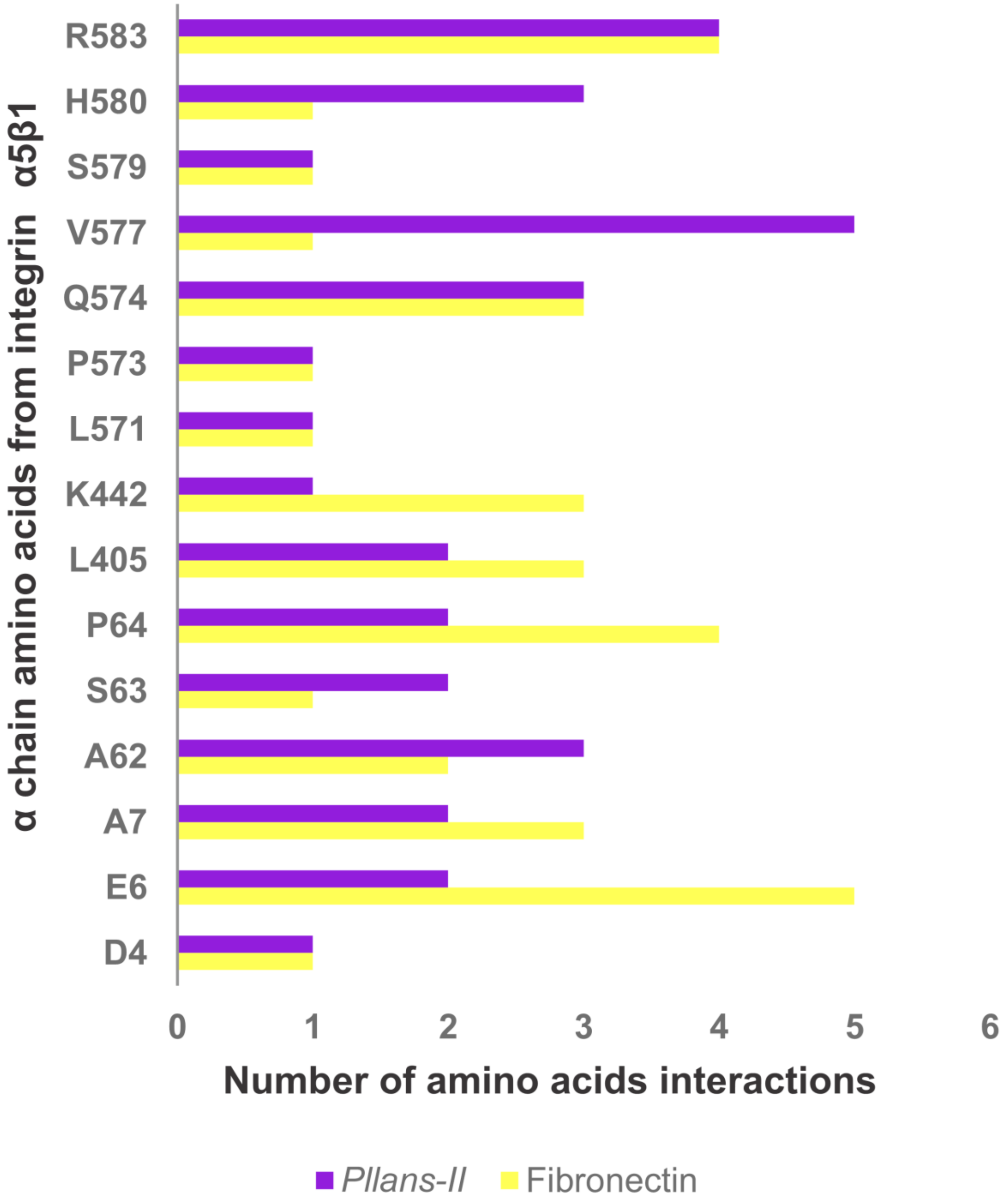

Figure S1. α5β1 integrin amino acid residues with potential for multiple interactions with *Pllans-II* amino acids (purple) and fibronectin amino acids (yellow).

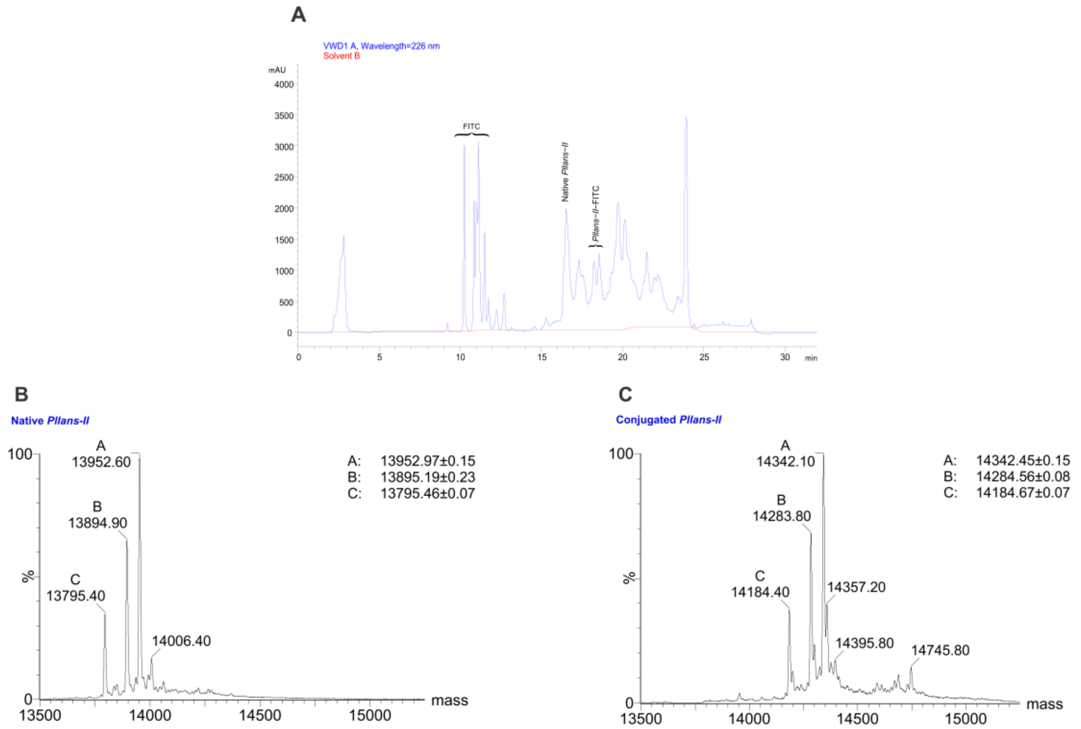

Figure S2: RP-HPLC and mass spectrometry analyses of *Pllans-II* conjugation with FITC. (**A**) RP-HPLC chromatographic profile of the FITC conjugation reaction. Peaks between retention times of 10 and 14 minutes correspond to FITC solution components. The peak with a retention time of 16.5 minutes corresponds to the *Pllans-II* protein that was not conjugated, while the peaks with a retention time between 18 and 18.6 minutes correspond to *Pllans-II* conjugated with FITC. (**B**) Mass spectrum of native *Pllans-II*, in which it is evident that the protein is present in three isoforms (**A-C**) that differ by 57 Da and 101 Da, respectively. (**C**) The mass spectrum of conjugated *Pllans-II* shows that the three isoforms differ from the native ones by approximately 389 Da, which is the mass of FITC.

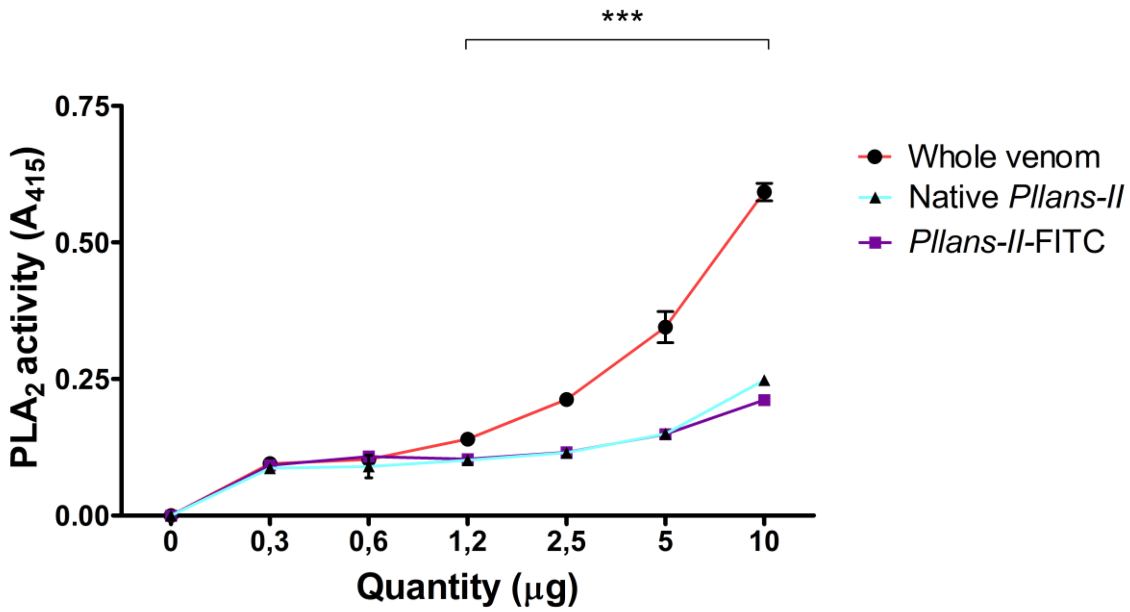

Figure S3. FITC-derivatized *Pllans-II* retains its enzymatic activity. Comparison of phospholipase A_2 activity of derivatized *Pllans-II* (*Pllans-II*-FITC, purple line), in contrast to native *Pllans-II* (light blue line) and complete venom (red line). The assay was carried out on NOBA monodisperse substrate. Data are expressed as mean ± SD, and procedures were developed in triplicate. Statistically significant differences are observed with *** $p < 0.001$.

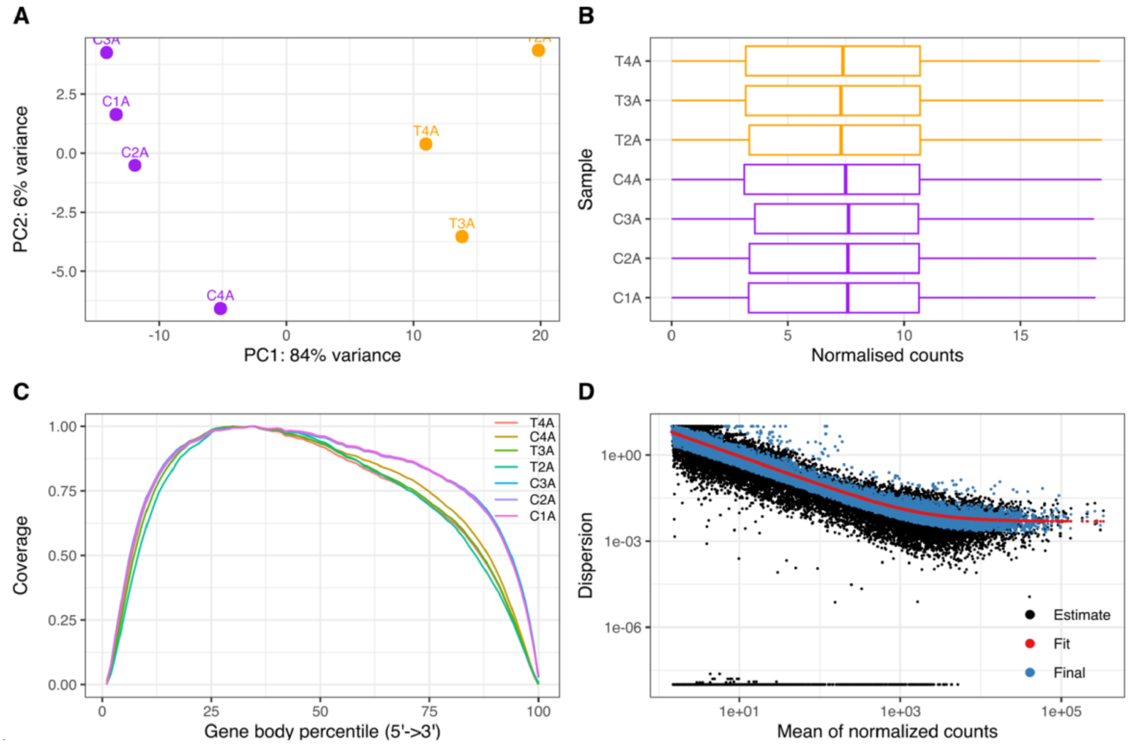

Figure S4. Quality control for transcriptomic analysis. (**A**) Principal component analysis (PCA). It is observed that the *Pllans-II* treatment explains 84% of the variation, while the intragroup variation represented 6%. The clustering pattern was as expected, and no potential outliers were observed for the samples. (**B**) Counting distributions. The normalized count distributions presented a similar gene expression profile, making them comparable for differential expression analysis. (**C**) Gene body coverage. A uniform distribution of the reads indicated the absence of sample degradation during sequencing. (**D**) Scatterplot and the mean of the normalized counts. The spread decreased as the mean of the normalized counts for each gene increased, indicating a good data set.
